# Draft Genome Sequence of Pseudomonas syringae RAYR-BL, a Strain Isolated from Natural Accessions of Arabidopsis thaliana Plants

**DOI:** 10.1128/mra.01001-21

**Published:** 2022-01-13

**Authors:** Isabel Fuenzalida-Valdivia, Maria Victoria Gangas, Diego Zavala, Ariel Herrera-Vásquez, Fabrice Roux, Claudio Meneses, Francisca Blanco-Herrera

**Affiliations:** a Centro de Biotecnología Vegetal, Facultad de Ciencias de la Vida, Universidad Andres Bello, Santiago, Chile; b Center of Applied Ecology and Sustainability (CAPES), Santiago, Chile; c ANID-Millennium Science Initiative Program-Millennium Institute for Integrative Biology (iBio), Santiago, Chile; d FONDAP Center for Genome Regulation, Centro de Biotecnologia Vegetal, Facultad de Ciencias de la Vida, Universidad Andres Bello, Santiago, Chile; e Laboratoire des Interactions Plantes-Microbes-Environnement, Institut National de Recherche pour l’Agriculture, l’Alimentation et l’Environnement, CNRS, Université de Toulouse, Castanet-Tolosan, France; University of Arizona

## Abstract

Here, we report the genome sequence of the P. syringae strain RAYR-BL, isolated from natural accessions of *Arabidopsis* plants. The draft genome sequence consists of 5.85 Mbp assembled in 110 contigs. The study of P. syringae RAYR-BL is a valuable tool to investigate molecular features of plant-pathogen interaction under environmental conditions.

## ANNOUNCEMENT

Pseudomonas syringae is a Gram-negative bacterium able to colonize plants and produce disease in the most economically important crop species (1). More than 60 pathovars have been recognized in the species (2), each with genetic characteristics improving their fitness to colonize particular plant species, even when potentially affecting different hosts (3). In 1991, Whalen et al. reported that P. syringae pv. tomato isolated from tomato plants can also infect the model plant Arabidopsis thaliana (4, 5). The wide range of genetic and technical tools offered by *Arabidopsis* and its capacity to host P. syringae pv. tomato as a pathogen result in a high-impact model system to dissect the physiological and molecular bases of the plant defense response (6, 7) and the mechanisms of bacterial pathogenicity.

**FIG 1:**
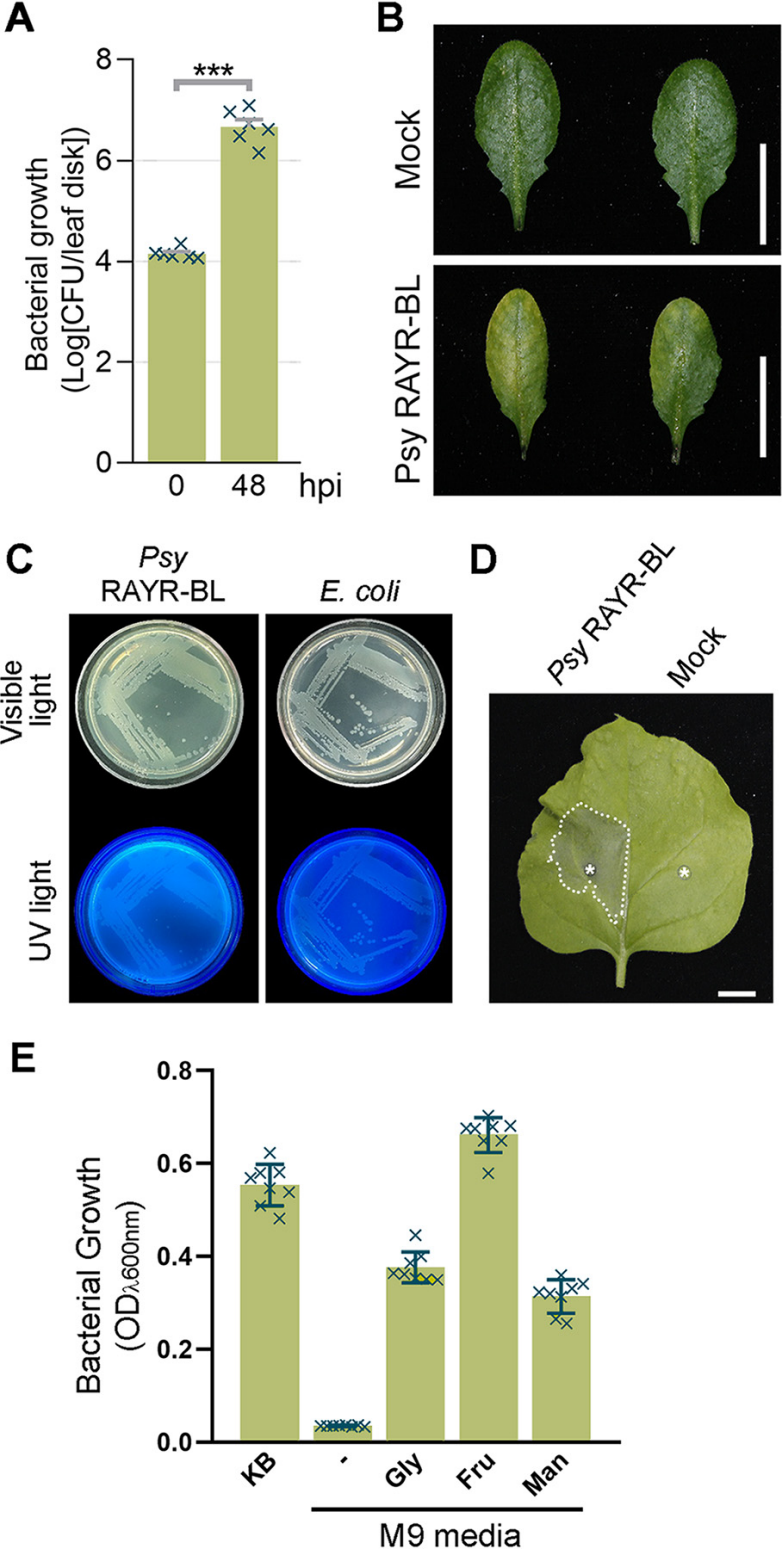
(A and B) Pseudomonas syringae RAYR-BL phenotypes. P. syringae RAYR-BL produces disease on the model plant Arabidopsis thaliana ecotype Columbia 0. Four-week-old *A. thaliana* (Col-0) plants were syringe-inoculated with P. syringae RAYR-BL (optical density at 600 nm [OD_600_], 0.1) or 10 mM MgCl_2_ as the control (mock), and they were maintained under normal growing conditions (16 h light/8 h dark cycle at 22°C, 100 mmol m^−2^ s^−1^, humidity ≈90%) until the leaves were sampled. (A) Bacterial proliferation is expressed as the mean of the log CFU/leaf disk ± standard deviation (SD) (*n* = 6). Disks (5 mm^2^) from inoculated leaves were cut using a punch immediately after inoculation (0 h postinfiltration [hpi]) and 48 hpi. Each disk was sampled from a different leaf from a different plant. The disks were ground on sterile MgCl_2_ and then plated on solid King’s B selective medium. The colonies were manually counted. Asterisks indicate statistical differences (*P* < 0.001) in an unpaired *t* test. (B) The plants inoculated with P. syringae RAYR-BL displayed a disease phenotype characterized by chlorosis after 72 hpi. Bar = 1 cm. (C) P. syringae RAYR-BL produces fluorescent molecules when it grows on King’s B medium. The bacteria P. syringae RAYR-BL and Escherichia coli were cultured on King’s B medium plates at 28°C. After 48 h the plates were photographed under UV and visible light. (D) P. syringae RAYR-BL produces disease symptoms on tobacco plants; 4-week-old plants were syringe-inoculated with P. syringae RAYR-BL (OD_600_, 0.01) on the left side of the leaf. As a control, MgCl_2_ (mock) was infiltrated on the right side. The asterisks indicate the inoculation site. The plants were maintained under normal growing conditions (16 h light/8 h dark cycle at 22°C, 100 mmol m^−2^ s^–1^, humidity ≈40%) for 48 hpi. Then the inoculated leaves were collected and photographed. The dotted line limits the leaf lesion. Bar = 1 cm. (E) The P. syringae RAYR-BL growth was evaluated on media supplemented with different carbon sources. The strain was incubated (28°C, 24 h, 180 rpm) on King’s B (KB) medium as a rich medium, M9 medium without carbon sources (–), or M9 medium supplemented with glycerol (Gly, 40 mM), fructose (Fru, 20 mM), or mannitol (Man, 20 mM). The data represent the mean of the OD_600_ ± SD (*n* = 8).

In 2018, Bartoli et al. isolated bacterial microorganisms from natural *Arabidopsis* accessions located in the south of France, identifying a new strain of Pseudomonas, P. syringae RAYR-BL, able to produce severe disease symptoms on different natural *Arabidopsis* accessions (8). This strain also infects *Arabidopsis* Col-0 plants, increasing the bacterial leaf population and inducing disease-associated phenotypic changes (Fig. 1). The study of the genome of the P. syringae RAYR-BL strain found in *Arabidopsis* plants will allow us to determine the pathogenicity mechanisms of species that naturally coexist and interact in the environment.

To study the genetic features of the P. syringae RAYR-BL strain, the bacteria were sent on semisolid medium from INRA-France from Fabrice Roux’s laboratory. The bacteria were expanded on liquid King’s B medium and stored at −80°C (15% glycerol). For DNA extraction, the stored bacteria were plated on King’s B medium, and then a single colony was grown on King’s B medium for 24 h at 28°C under shaking conditions. Genomic DNA isolation was performed using a Qiagen DNeasy blood and tissue kit. Then, 100 ng of the isolated DNA was used in a TruSeq DNA sample preparation kit (Illumina, Inc., USA). Whole-genome sequencing of P. syringae RAYR-BL was performed on a MiSeq platform at the Plant Biotechnology Center of Universidad Andres Bello, Chile. We obtained 2,723,120 paired-end reads of 300-bp length (2 × 300 bp). Default parameters were used for all software unless otherwise specified. Quality assessment of the reads was done using FastQC v0.11.7 (9). Removal of low-quality reads and adapter content was performed with Trimmomatic v0.38 (SLIDINGWINDOW:10:25 MINLEN:50) (10). The filtered reads were assembled *de novo* using SPAdes v3.11.0 (--careful -k 77, 89, 99) (11), and the draft assembly metrics were obtained with QUAST v5.1 (12). Contigs shorter than 200 bp were discarded, and those remaining were ordered against the reference Pseudomonas syringae DC3000 genome (13) using the Mauve Contig Mover software v2.4 (14).

The resulting draft genome sequence of P. syringae RAYR-BL consists of 5,853,599 bp assembled in 110 contigs, with an average G+C content of 58.98%, an *N*_50_ value of 193,008 bp (largest contig, 1,064,144 bp), and an *L*_50_ value of 8 contigs. The completeness of the draft genome assembly was assessed with a BUSCO (Benchmarking Universal Single-Copy Orthologs) v4.0.5 test (15), showing a completeness of 98.6% using the *Pseudomonadales* (OrthoDB v10) as a reference orthologue set. The genome annotation was made using Prokka v1.12 (--addgenes --gram neg --rfam --usegenus --genus Pseudomonas, with the Pseudomonas database created with all the complete Pseudomonas genome sequences found in the NCBI assembly database) (16), which predicted 5,188 genes, 5,053 coding sequences, 6 rRNA genes, and 57 tRNA genes. A phylogeny tree based on the citrate synthase (cts) sequence reveals that P. syringae RAYR-BL belongs to the P. syringae complex (8). Even when average nucleotide identity (ANI) values obtained with PyANI v0.2 (17) using ANI based on BLAST (ANIb) analysis reported that the best match corresponds to Pseudomonas tremae (88.29%), comparison with other P. syringae strains such as P. syringae pv. oryzae strain 1_6 and P. syringae DC3000 show similar ANIb values of 87.7% and 87.4%, respectively. To clarify the taxonomic status, further phenotypic analysis demonstrates that P. syringae strain RAYR-BL shares the following important features with the P. syringae species, unlike *P. tremae*: (i) the production of fluorescent molecules when it grows on King’s B medium (Fig. 1C) (18), (ii) the induction of disease phenotype on the host plant Nicotiana benthamiana (Fig. 1D) (19), and (iii) the capacity to metabolize and grow on glycerol, fructose, and mannitol as carbon sources (Fig. 1E) (18).

### Data availability.

This whole-genome shotgun project for P. syringae RAYR-BL has been deposited at DDBJ/ENA/GenBank under the accession number JAHZNS000000000. The version described in this paper is version JAHZNS010000000. The raw sequencing reads for this project can be found under SRA accession number SRR15318771. The BioProject accession number is PRJNA750763.

## References

[1] Xin X-F, Kvitko B, He SY. 2018. Pseudomonas syringae: what it takes to be a pathogen. Nat Rev Microbiol 16:316–328. doi:10.1038/nrmicro.2018.17. 29479077 PMC5972017

[2] Baltrus DA, McCann HC, Guttman DS. 2017. Evolution, genomics and epidemiology of Pseudomonas syringae: challenges in bacterial molecular plant pathology. Mol Plant Pathol 18:152–168. doi:10.1111/mpp.12506. 27798954 PMC6638251

[3] Wroblewski T, Caldwell KS, Piskurewicz U, Cavanaugh KA, Xu H, Kozik A, Ochoa O, Mchale LK, Lahre K, Jelenska J, Castillo JA, Blumenthal D, Vinatzer BA, Greenberg JT, Michelmore RW. 2009. Comparative large-scale analysis of interactions between several crop species and the effector repertoires from multiple pathovars of Pseudomonas and Ralstonia. Plant Physiol 150:1733–1749. doi:10.1104/pp.109.140251. 19571308 PMC2719141

[4] Whalen MC, Innes RW, Bent AF, Staskawicz BJ. 1991. Identification of Pseudomonas syringae pathogens of Arabidopsis and a bacterial locus determining avirulence on both Arabidopsis and soybean. Plant Cell 3:49–59. doi:10.2307/3869199. 1824334 PMC159978

[5] Goode MJ, Sasser M. 1980. Prevention: the key to controlling bacterial spot and bacterial speck of tomato. Plant Dis 64:831. doi:10.1094/PD-64-831.

[6] Kim MG, Kim SY, Kim WY, Mackey D, Lee SY. 2008. Responses of Arabidopsis thaliana to challenge by Pseudomonas syringae. Mol Cells 25:323–331. 18483469

[7] atagiri F, Thilmony R, He SY. 2002. The Arabidopsis Thaliana-Pseudomonas Syringae interaction. Arabidopsis Book 1:e0039. doi:10.1199/tab.0039. 22303207 PMC3243347

[8] Bartoli C, Frachon L, Barret M, Rigal M, Huard-Chauveau C, Mayjonade B, Zanchetta C, Bouchez O, Roby D, Carrère S, Roux F. 2018. In situ relationships between microbiota and potential pathobiota in Arabidopsis thaliana. ISME J 12:2024–2038. doi:10.1038/s41396-018-0152-7. 29849170 PMC6052059

[9] Wingett SW, Andrews S. 2018. FastQ screen: a tool for multi-genome mapping and quality control. F1000Res 7:1338. doi:10.12688/f1000research.15931.1. 30254741 PMC6124377

[10] Bolger AM, Lohse M, Usadel B. 2014. Trimmomatic: a flexible trimmer for Illumina sequence data. Bioinformatics 30:2114–2120. doi:10.1093/bioinformatics/btu170. 24695404 PMC4103590

[11] Bankevich A, Nurk S, Antipov D, Gurevich AA, Dvorkin M, Kulikov AS, Lesin VM, Nikolenko SI, Pham S, Prjibelski AD, Pyshkin AV, Sirotkin AV, Vyahhi N, Tesler G, Alekseyev MA, Pevzner PA. 2012. SPAdes: a new genome assembly algorithm and its applications to single-cell sequencing. J Comput Biol 19:455–477. doi:10.1089/cmb.2012.0021. 22506599 PMC3342519

[12] Gurevich A, Saveliev V, Vyahhi N, Tesler G. 2013. QUAST: quality assessment tool for genome assemblies. Bioinformatics 29:1072–1075. doi:10.1093/bioinformatics/btt086. 23422339 PMC3624806

[13] Buell CR, Joardar V, Lindeberg M, Selengut J, Paulsen IT, Gwinn ML, Dodson RJ, Deboy RT, Durkin AS, Kolonay JF, Madupu R, Daugherty S, Brinkac L, Beanan MJ, Haft DH, Nelson WC, Davidsen T, Zafar N, Zhou L, Liu J, Yuan Q, Khouri H, Fedorova N, Tran B, Russell D, Berry K, Utterback T, Van Aken SE, Feldblyum TV, D’Ascenzo M, Deng W-L, Ramos AR, Alfano JR, Cartinhour S, Chatterjee AK, Delaney TP, Lazarowitz SG, Martin GB, Schneider DJ, Tang X, Bender CL, White O, Fraser CM, Collmer A. 2003. The complete genome sequence of the Arabidopsis and tomato pathogen Pseudomonas syringae pv. tomato DC3000. Proc Natl Acad Sci U S A 100:10181–10186. doi:10.1073/pnas.1731982100. 12928499 PMC193536

[14] Rissman AI, Mau B, Biehl BS, Darling AE, Glasner JD, Perna NT. 2009. Reordering contigs of draft genomes using the Mauve aligner. Bioinformatics 25:2071–2073. doi:10.1093/bioinformatics/btp356. 19515959 PMC2723005

[15] Simão FA, Waterhouse RM, Ioannidis P, Kriventseva EV, Zdobnov EM. 2015. BUSCO: assessing genome assembly and annotation completeness with single-copy orthologs. Bioinformatics 31:3210–3212. doi:10.1093/bioinformatics/btv351. 26059717

[16] Seemann T. 2014. Prokka: rapid prokaryotic genome annotation. Bioinformatics 30:2068–2069. doi:10.1093/bioinformatics/btu153. 24642063

[17] Pritchard L, Glover RH, Humphris S, Elphinstone JG, Toth IK. 2016. Genomics and taxonomy in diagnostics for food security: soft-rotting enterobacterial plant pathogens. Anal Methods 8:12–24. doi:10.1039/C5AY02550H.

[18] Gardan L, Shafik H, Belouin S, Broch R, Grimont F, Grimont PAD. 1999. DNA relatedness among the pathovars of Pseudomonas syringae and description of Pseudomonas tremae sp. nov. and Pseudomonas cannabina sp. nov. (ex Sutic and Dowson 1959). Int J Syst Bacteriol 49:469–478. doi:10.1099/00207713-49-2-469. 10319466

[19] Islam MN, Ali MS, Choi SJ, Park Y-Il, Baek KH. 2019. Salicylic acid-producing endophytic bacteria increase nicotine accumulation and resistance against wildfire disease in tobacco plants. Microorganisms 8:31. doi:10.3390/microorganisms8010031. PMC7022923 31877906

